# “Slipping Through the Net”: Support Workers’ Perspectives on Domestic Violence Services for Minority Ethnic Women

**DOI:** 10.1177/10778012261440249

**Published:** 2026-04-13

**Authors:** Majka Ryan, Noreen Heraty

**Affiliations:** 1 Department of Work and Employment Studies, 8808 Kemmy Business School, University of Limerick, Limerick, Ireland

**Keywords:** domestic violence support service provision, minority ethnic women victim-survivors of domestic violence, intersectionality, role-taking

## Abstract

To advance more equitable access to supports for all victim-survivors of domestic violence (DV), this article evidences the complexity of Domestic Violence Support Service (DVSS) provision to minority ethnic women in Ireland. Drawing on semi-structured interviews with DVSS providers, it shows how compounded structural and intersectional challenges affect support quality and limit providers’ capacity to understand and identify with these women's experiences. The article reveals providers’ approaches and identifies systemic, institutional, and practical barriers to effective DV support. Using intersectionality and role-taking, the article offers a holistic understanding of structural and practical shortcomings that adversely affect both minority ethnic women and support workers.

## Introduction

The Irish Court Service emphasizes that domestic violence (DV):

includes—but is not limited to—any act or threat of physical, sexual, or emotional violence, or coercive control (…). Violent acts or threats can be made by relatives such as parents, adult children, or grandparents, as well as by intimate partners—current or former spouses, civil partners, cohabiting partners, your child's other parent, or unmarried partners (https://www.courts.ie/).

According to Safe Ireland (SI), “domestic abuse and coercive control is a persistent and deliberate pattern of behavior by an abuser over a prolonged period of time designed to achieve obedience and create fear” (https://www.safeireland.ie/). The Government of Ireland (2022, p. 11) in its Third National Strategy on Domestic, Sexual and Gender-Based Violence (DSGBV) 2022–2026 defines DV as “a human rights abuse and a form of gender-based violence with its roots in gender inequality.” Although national statistics on the prevalence of DV in Ireland are unavailable (Kavanagh & Fassbender, 2024), it is acknowledged that DV in Ireland, as in many parts of the world, is a “long-standing, large-scale national social problem” (SI, 2023, p. 8). Data drawn from individual agencies and Domestic Violence Support Services (DVSSs) paint a grim picture. According to FRA et al. (2024), 40.7% of women in Ireland have experienced physical violence or threats and/or sexual violence in their lifetime, while 35% of women have experienced such violence from an intimate partner. Women's Aid (2025) which is the leading Irish DVSS organization, received the highest contact rates in the organization's 50-year history in 2024, with 41,432 disclosures involving women and 5,333 involving children in 2024. This represented a 17% increase in disclosures of DV reported in 2023 (https://www.womensaid.ie). Femicide is also on the rise in Ireland; in 2021 a staggering 52% of all murders were of women (An Garda Síochána, 2022). There is currently no systematic collection of ethnically disaggregated data across the Irish justice system (Government of Ireland, 2023).

A core principle of the 2030 Agenda for Sustainable Development is to “Leave No One Behind.” Moreover, Sustainable Development Goal 5, Target 5.1 aims to “end all forms of discrimination against women and girls everywhere” (https://unstats.un.org). To generate in-depth insights towards achieving equal and equitable access to supports and better outcomes for *all* victim-survivors of DV, in this article we examine the existing DVSS landscape for minority ethnic women in Ireland. Minority ethnic women represent a distinct group of service users within DVSS. These survivors are marginalized in multiple ways and face heightened structural violence stemming from intersecting systems of discrimination. In addition to presenting with a myriad of emotional and physical health issues (Parson et al., 2014), they tend to be legally disempowered (Mogulescu, 2020), and encounter numerous additional barriers to seeking help, including financial dependence, stigma, culturally specific understandings of abuse within their communities and the associated ostracization (see Erez & Britz, 2006).

Abuse can be embedded within national systems of authority resulting in institutional coercive control. Sokoloff and Dupont (2005, p. 44) argued that “the lack of adequate institutional support in the form of social services and public housing as well as the intrusions and coercive controls by the state and its agencies (e.g., welfare) is another level of violence experienced by battered women.” Godsey and Robinson (2014) further noted that formal systems, such as welfare institutions and immigration services, can maintain the abuser's power over his victim by restricting her autonomy. In addition to systemic barriers and intersectional vulnerabilities, minority ethnic women are subject to institutional coercive control, which can push some to the margins of society (Sokoloff & Dupont, 2005). Many of these women have precarious legal situations: their visas may have expired; they may be currently applying for or have been refused international protection; they may have been trafficked; they may be undocumented, or they may be international students with strictly conditional, limited, or no legal residency rights (Di Matteo & Scaramuzzino, 2022). For some, their precarious immigration status means that their residency, and access to social security, is contingent on their maintaining a relationship with the perpetrator. Women who join intimate partners through family reunification are often socially isolated. Day and Gill (2020) found that the intersections of gender, class, “race” and immigration status have a major impact on the effectiveness of the supports provided to DV victim-survivors, while McIlwaine (2023) argued that the struggles of migrant women victim-survivors of DV to access support are exacerbated by their insecure immigration status, language competency, lack of information and social networks. Immigration status is weaponized by perpetrators to enforce dependency and silence (Voolma, 2018), while institutional policies compound economic and social isolation by constraining access to welfare and legal remedies. In sum, despite recommendations to “remove all barriers to accessing support for migrant women experiencing gender-based violence” (Government of Ireland, 2020, p. 23), these women's precarious immigration status and legal disempowerment still render them heavily reliant on their intimate partners, families, and diasporas, which significantly increases safety risks (Reilly, 2023), hinders their ability to seek support and heightens their vulnerability (Erez & Britz, 2006).

Understanding DV, and responses to it, requires fuller recognition of the inequality perpetuated by systemic and institutional violence, including sexism, racism and cultural marginalization (Essue et al., 2025). This matrix of domination (Hill-Collins, 2000), or intersecting forms of oppression, compounds the experience of DV and affects both victim-survivors’ help-seeking behaviors and the quality of the supports that they receive (Gill & Anitha, 2023).

This article presents a narrative account of DVSS provision to minority ethnic women in Ireland to demonstrate how the intersecting complexities of the lived experiences of victim-survivors are being addressed by DVSS workers. We first present a brief overview of DVSS in Ireland to contextualize our study. We frame our research within a conceptual framework that incorporates intersectionality and role-taking perspectives. We argue that examining the landscape of DVSS provision through these combined lenses offers a more holistic and nuanced understanding of both structural shortcomings within the current support ecosystem, and the attendant implications for how these DVSS workers perform their roles. Finally, based on our findings, we present recommendations for policy, practice, and future research.

## The DVSS Landscape in Ireland

Originating from the grassroots women's movement, DVSS in Ireland emerged in the 1970s primarily via voluntary groups that publicly drew attention to women's oppression. The 50 years of subsequent development of Ireland's DVSS resulted in a vast network of organizations, employees, and specialist staff. Today, in collaboration with other agencies, such as An Garda Síochána (the national police service), legal representatives and court services and community and voluntary organizations, DVSSs provide holistic interventions for victim-survivors of DV. As described by SI (2023, p. 7), “each local organization delivers various combinations of safe accommodation, support/referral services, including crisis helplines, one-to-one emotional and therapeutic support, practical support, information, advocacy, accompaniment and referral in relation to legal supports, housing, finance, health, education, employment, [and] child protection.” Often fully or partially funded by the State, DVSSs play a key national role in preventing and addressing DV, helping victim-survivors to escape abuse and rebuild their lives.

O'Sullivan (2023) argues that the contemporary approach of the Irish State to violence against women must be interpreted through the lens of its historical foundations, which are deeply influenced by traditional Catholic doctrines and patriarchal social structures. Ireland has a long history of protecting men who abuse women and children, as evidenced, for example, by its handling of the Magdalene Laundries. Research shows that these State-enabled gender power imbalances persist today (Kavanagh & Fassbender, 2024) and are reflected in “structural refusals to formally address both implicit patriarchal, along with patterned perpetrator weaponization of, ‘protective’ systems across justice, homelessness and housing, social protection, health and finance,” resulting in the “structural erasure of DSGBV victim-survivors’ realities (SI, 2023, p. viii).”

As part of the DVSS ecosystem, DV refuges in Ireland play a key role in offering women not only a safe space away from immediate danger, but also an opportunity to reflect on their situation, make decisions, and connect with support services. However, there is a severe shortage of DV refuges across the country (O’Halloran, 2024). In addition, the Irish housing crisis means that those who are “lucky” enough to secure refuge accommodation have nowhere to move on to, causing anxiety and undermining these women's sense of safety and ability to make long-term plans (SI, 2023). Moreover, victim-survivors who co-own their home are not entitled to social housing. Women who leave an abusive home, whether privately owned or a social tenancy, are penalized by the State, “via blacklisting from housing supports for one year” for “making themselves” homeless (Kavanagh & Fassbender, 2024, p. 15). Finally, the lack of affordable accommodation can force victim-survivors to quit work, “as earned income puts them above designated thresholds for legal and housing supports, but entraps them in poverty” (Kavanagh & Fassbender, 2024, p. 10).

The function of DVSS is embedded within the broader network of national and European legal and welfare systems. In recent years, Ireland has made significant legal and policy advancements which reflects a growing commitment by the State to promote equality and protect women's rights. These include its Zero Tolerance Third National Strategy on Domestic, Sexual and Gender-Based Violence (DSGBV), and the establishment of a statutory agency, CUAN, with responsibility for coordinating and driving this strategy. The strategy commits to addressing the multidimensional nature of inequality, with a particular focus on its intersection with gender and migration—factors that heighten vulnerability among minority ethnic victim-survivors. In so doing it aims to provide social safety nets for *all*.

Since 2019, Ireland has been a signatory to the Istanbul Convention (Council of Europe Convention on Preventing and Combating Violence against Women and Domestic Violence). However, a report by the Rape Crisis Network Ireland (RCNI) and SI (2022, p. 5) noted that “there remains a significant gap between *de jure* and *de facto* implementation of the Istanbul Convention.” In practice, not all female victim-survivors of DV are equally supported or protected by the State. Intersectionality effects are far reaching because, as noted by the Irish Government, “social biases influence how society perceives survivors of violence, and stereotypes often create barriers to care and assistance, often coupled with women's individual and family social position, and negatively affecting help-seeking patterns” (Government of Ireland 2022, p. 12). The social group that comprises minority ethnic women victim-survivors is especially prone to intersectional disadvantage, comprising as it does Traveller and Roma women, refugee and asylum-seeking women, victims of torture, older women, women with disabilities, women that identify as LGBTQI+, and women who are trafficked, undocumented or precariously resident in Ireland. Almost two decades ago, Allen and Foster (2007) stressed that we must recognize the considerable and diverse nature of DV that minority ethnic women experience. However, they further noted that “common experiences can be identified among ethnic minority women in domestic violence situations and that responsive service provision requires an awareness of, and sensitivity to, such experiences” (Allen & Foster, 2007, p. 4).

Recent evidence from both Women's Aid (2025) and Kavanagh and Fassbender (2024) highlight the growing demand for DVSS and report the extreme isolation experienced by minority ethnic women, exacerbated by language barriers and their limited awareness of available supports and legal protections in Ireland. In addition, migrant victim-survivors are primarily categorized based on their immigration status. Residency rules, particularly the Habitual Residence Condition derived from the European Directive 2004/38/EC, pose barriers to support and create significant challenges in service provision. Many of these women have no one to turn to and some are so terrified and confused that they are afraid to engage even with their own legal representatives (Kavanagh & Fassbender, 2024).

DVSS work involves providing empowering, client-centered services that encourage the victim-survivor's voice and enable her capacity to make her own decisions and choices. Thus, the approach to victim-survivors is flexibly adapted according to their individual needs. As Kulkarni et al. (2012, p. 98) emphasized, “domestic violence service providers must have a broad sense of their mission to address women's needs in their totality, respecting individual survivors’ perceptions of their needs and preferred solutions.” In addition, their work context can be highly stressful, emotionally charged and personally demanding (Molloy 2019). They interact with women that are physically and emotionally hurt, listen to their dreadful stories, and frequently witness their returning to the perpetrators and to further abuse. “Service providers experience high levels of cumulative stress from direct and indirect exposure to trauma while working closely with trauma survivors in a culture that tends to minimize or deny the existence of domestic violence, and therefore, provides limited resources for addressing the aftermath of such violence” (Kulkarni et al., 2013, p. 115). The report by SI (2023) highlighted that DVSS frontline staff are sensitive to the vulnerabilities that minority ethnic women experience and acknowledged that they must be adequately resourced to enable them to effectively assist all victim-survivors. The report specifically called attention to DVSS staff wellbeing to ensure that “the response to DSGBV will not be diluted due to disillusionment, burnout or economic necessity” (SI, 2023, p. iii).

Having briefly outlined the landscape of DVSS in Ireland, we turn now to a consideration of the conceptual perspectives, namely intersectionality and role-taking, that we used to frame our study.

## Intersectionality

Some 20 years ago, Sokoloff and Dupont (2005) acknowledged the growing scholarship on intersectionality at that time that challenged the primacy of gender as an explanatory model of DV and called for more research on the structural inequalities that contribute to the incidences of abuse that are experienced in intersectional ways. In the intervening years, intersectionality perspectives have grown in application, particularly within women's and gender studies (O’Connell et al., 2023), while research on sexual violence has increasingly acknowledged inequality, not only on the basis of gender, but also along lines of race/ethnicity, class, sexuality, age, ability status, citizenship status, and nationality (Armstrong et al., 2018).

Crenshaw's (1991) classic work on intersectionality explicitly cites immigration status (in addition to racial identity) as having a significant influence on the experiences of DV among African American women in the United States. Her work underscored the ways in which social categories, including but not limited to race, class, ability, gender, and sexuality, interact to shape their experiences. Hill-Collins (2000, p.43) referred to “the matrix of domination” in which multiple systems of oppression interlock to create a complex web of power relations that shape individual realities and experiences. Homan et al. (2021) later argued that this imbalance of power within societal structures, systems, and processes particularly and detrimentally impacts women with marginalized identities.

Interest in giving voice to the experiences of minority ethnic women victim-survivors of DV is growing, leading Bastia (2014, p.240) to describe them as potentially “the new quintessential intersectional subject.” Misztal (2011) argued for better ways of capturing specific socio-cultural challenges faced by women from refugee and immigrant communities, while Segrave's (2017) research provided a detailed analysis of the ways in which migration systems can contribute to and sustain power inequality for non-citizen women experiencing family violence. Intersectionality frames disadvantage not as a simple sum of separate factors but as a dynamic interplay between factors. Violence does not have a single cause and violence against women must account for the intersecting factors that shape the lived realities of affected women (Lockhart & Danis, 2010). More recently, Kulkarni (2019) emphasized a need for a more intersectional DVSS orientation, one that considers survivors’ multiple identities and priorities beyond victimization and safety.

Burman et al. (2004, p. 332) argued that “discourses of service provision to minoritized women” can be informed by “a homogenized absence; or alternatively as a pathologized presence.” On one hand, acknowledging of intersectionality and cultural differences is the key for support workers to understand individual experiences of DV and to respond effectively to women's needs. On the other hand, however, support workers must avoid overemphasizing cultural difference or using the prevalence of DV to excuse abuse for “cultural reasons” (Burman et al., 2004, p. 332). Misztal (2011) proposed that minority ethnic women's vulnerability should be considered less as a pre-existing status or categorization but rather in terms of the ways in which social and political factors create and/or intensify the risks for these women. This perception of vulnerability from a risk orientation is gaining support. Kapur et al. (2017) found that inattention, or indifference to migration specific risks, both at individual and structural levels, heighten vulnerabilities among such women, while Maher and Segrave (2018) similarly argue that risk intensifies with heightened dependence, uncertainty or fear.

This interpretation of vulnerability from a risk perspective is compelling, and we argue that it can also be applied to those who provide support to minority ethnic women–in this case, the support workers–as their work is potentially damaging. Noting that it is variously and overlappingly depicted as secondary traumatic stress (STS), compassion fatigue (CF), vicarious traumatization (VT), and burnout (BO), Kanno and Giddings (2017) describe secondary exposure to trauma as the effects of exposure of repeatedly hearing the details of traumatic events experienced by others and being unable to respond in a typical fashion. Rather, their job requires them to sit calmly, always listen intently and project empathy even when they are hearing highly distressing content. While beyond the scope of this article, this application of vulnerability is worth exploring as it reflects a growing concern to better understand the impact of providing DV care on the providers themselves (Britt et al., 2021; Lundy & Crawford, 2025).

## Role-Taking

Having established how intersectionality affects DV service provision, we now move to consider role-taking as a particularly useful framework to investigate the social interactions between the support worker and the DV victim-survivors.

First described by Mead (1934), role-taking refers to the capacity of an individual to learn how others respond to them, and to learn to place themselves in the position of the other and to see the world as the other sees it. Forte (1998) described role-taking as a mechanism in interpersonal communication whereby one individual takes the role/perspective/attitude of the other or empathizes with that other to create a shared world with them. More simply, role-taking is a capacity to put yourself in someone else's shoes (Groggel et al., 2022). This ability to understand the perspective of others is a key underlying feature of successful social interactions and one which enables coordination and problem solving.

Role-taking can be seen as a critical and necessary component of DVSS work because, as Cast (2004) suggested, individuals who feel they are able to understand the other person's perspective should be more likely to behave in ways that support the other person, and less likely to criticize and alienate them. Forte (1998) noted that role-taking, especially empathy or affective role-taking, is a useful means to recount the ways in which support workers attempt to give voice to marginalized members of society. Role-taking begins with the first interaction when early impressions and expectations are formed. During the formation of the relationship, support workers consciously work to learn how their clients are likely to respond to them and, as they build the relationship, they start to anticipate how they will react and respond to them and alter their behavior according to what they feel is required to best support the client. Recognizing that society's structural inequalities are reproduced in helping interactions, Forte (1998) noted that institutional and situational contingencies can influence the accuracy of role-taking and thus cautioned role-takers to be vigilant of the power differential that exists between the support worker and the service user. The service user's historic experience of feeling powerless may render them especially sensitive to cues interpreted as anger or criticism or threat and lead them to withdraw from treatment.

Groggel et al. (2022) identify two key components of role-taking namely perspective-taking (cognitive) and empathic role-taking (affective). The cognitive process involves the support worker anticipating what the service user needs from them and reading cues both from what they say as well as their body language. This then filters into their own responses and may result in changes to what they themselves say or do in order to make the client as comfortable as possible. Empathic role-taking has an affective component (Davis & Love, 2017). It involves not only trying to understand the service user's perspective but also being moved by their experiences and in some cases sharing their feelings and emotions. This suggests a deeper and more effortful form of role-taking. Groggel (2023) observed that while empathic role-taking is less common than cognitive role-taking, it is more likely to occur where workers share similar past experiences or social status with the victim-survivor. Other variables, including the organizational culture and supports are important in terms of mitigating the impact of role-taking. Reflecting overall on her findings, Groggel (2023, p. 70) concluded that “role-taking is simultaneously an antidote and poison–necessary for clients but potentially detrimental to workers’ well-being.”

Having outlined the conceptual framing of our research, we turn now to the core focus of our research. Our overall research question was to explore how intersectional identities and structural inequalities shape barriers to DVSSs, and influence support workers’ approaches to minority ethnic women victim-survivors in Ireland. Accordingly, three specific objectives guided our study: (a) To identify any barriers in DVSS provision for minority ethnic women victim-survivors; (b) To explore how these barriers are shaped by intersecting social identities and structural inequalities; (c) To examine how intersectional vulnerabilities influence support workers’ approaches to and interaction strategies for providing DV services. The conceptual framework adopted for the study enabled a more holistic and systematic investigation of the support landscape. An intersectionality perspective helped us better understand the barriers to DVSSs for these women. It also allowed us to uncover the complexities that arise in DVSS provision within the context of diverse cultural needs and revealed the perceived adequacy (or otherwise) of the available services. The incorporation of role-taking provided insight into the individual experiences of service providers to minority ethnic women. In so doing, it uncovered how support workers approach these women and illustrated the impact of intersectionality on their professional practice.

## Methodology

Our research, based on in-depth, semi-structured interviews, drew on the lived experiences of a sample of support workers involved in frontline DVSS provision to minority ethnic women in Ireland. Adopting an interpretive approach, we explored how DVSS workers perceive, interpret, and respond to the diverse needs of these women. Our choice of sample helps address concerns that the voices of those on the front lines of service delivery are not always included. We acknowledge that this concern similarly applies to the voices of those who experience DV directly (Mengo et al., 2023). However, given that this research focused on the experiences of support workers in order to capture the challenges and negotiations involved in service provision, victim-survivors were not consulted. This decision was also made in recognition of the risk of re-traumatizing vulnerable groups (Ellsberg et al., 2001).

Purposeful sampling began upon receipt of ethics approval and aimed to capture a broad range of experiences in providing DVSSs. To enable cross-sectoral analysis, we targeted participants who would represent mainstream DVSSs, as well as immigrant support organizations and voluntary groups. As experts in migration-related matters, immigrant support organizations were included to provide insights about the varied legal constraints on service provision that DVSS workers must overcome when assisting minority ethnic women. Additionally, voluntary groups possess deep understanding of the communities that they serve. Thus, rather than focusing solely on mainstream DVSSs, we examined the cross-sectoral experiences of service provision to this cohort of women (Burman et al., 2004).

Recruitment of participants was aided by our DV community research partner organization. Its understanding of the DVSS landscape, combined with its nationwide network, significantly aided in identifying participants. It distributed our research invitation via email to its contacts, inviting participation or requesting that the invite be shared with other potentially relevant participants in their contact group. The latter snowballing strategy was similarity utilized with interview participants to aid purposive sampling (Scott, 2002).

A total of 23 invitations were issued to potential participants across the Republic of Ireland, of which 17 were accepted. To ensure participants had relevant knowledge and experience, only those with at least 6 months’ experience in DVSS provision were invited to interviews, and the final sample consisted of support workers with between 18 months and 15 years’ relevant experience. Characteristics of the participants are presented in Table 1.

**Table 1.** Participant Characteristics.

Pseudonym	Type of Organization	Position	Experience in DVSS	Work Mode
Patty	DVSS	Support Worker	1.5 years	Full-time Worker
Catherine	DVSS	Support Worker	8 years	Full-time Worker
Grace	DVSS	Coordinator	6 years	Full-time Worker
Samantha	Migrant Support Organization	Support Worker	12 years	Full-time Worker
Kate	DVSS	Support Worker	2 years	Full-time Worker
Caroline	DV Refuge	Refuge Manager	9 years	Full-time Worker
Sarah	DV Refuge	Refuge Coordinator	9 years	Full-time Worker
June	DVSS	Migrant Community Development Worker	8 years	Full-time Worker
Vicky	Migrant's Rights Organization	Legal Officer	3 years	Full-time Worker
Lorraine	DVSS	Deputy Director	6 years	Part-time Worker
Rachel	DVSS	Support Liaison Officer	7 years	Full-time Worker
Monica	Migrant Support Organization	Advice and Information Officer	7 years	Full-time Worker
Mary	Migrant Support Organization	Support Officer	1 year	Full-time Worker
Deirdre	Migrant Support Organization	Co-Founder/Leader	5 years	Part-time Volunteer
Ella	DV Refuge	Migrant Support Worker	1.5 years	Full-time Worker
Amy	Currently Non-affiliated	Support Volunteer	15 years	Part-time Volunteer
Laura	DVSS	Outreach Support Worker	6 years	Full-time Worker

The interviews took place between August and October 2024. All interviews were conducted online and video-recorded using the Microsoft Teams recording function. Each interview lasted between 60 and 75 minutes. All participants in the interviews were women, ranging in age from 28 to 69 years. Eight participants worked in mainstream DVSSs, 5 in migrant support organizations, 3 in DV refuges, and 1 was an unaffiliated volunteer at the time of the interview. Notably, 2 of the 17 participants were providing DVSS on a voluntary basis. Most participants held roles involving direct DVSS, 4 held supervisory or managerial positions, and 1 was a DV legal support worker.

Given the sensitive nature of the subject matter, participants were informed in advance of the purpose of the research and the voluntary nature of their participation. A research invitation letter, consent form, and research privacy notice explaining the research aims, requirements, withdrawal and privacy rights was sent to participants at least one week in advance of data collection. As an additional safeguard, the research information letter directed participants to MyMind (mymind.org), an organization that provides a nationwide therapist directory, and to Women's Aid 24-hour National Freephone Helpline (1800 341 900), should they feel in any way impacted by the interviews.

The data collection process rigorously adhered to data protection principles as outlined by the authors’ University's GDPR policy and was managed to the highest standards of security and confidentiality. From the outset, all participants were made aware of how their data would be used, and what personal data would be collected. Transcripts generated by Teams were anonymized immediately after each interview. Anonymized transcripts have been securely stored in encrypted formats within a dedicated, GDPR-compliant, OneDrive folder accessible only to the authors. The collected data will be retained for 7 years and subsequently destroyed by the first author.

All interviews were conducted by the first author, who has undertaken specialized training in understanding and responding to domestic abuse. They were mindful and observant of the participants through the interviews to ensure they were comfortable with the questions being asked and reassured that they did not need to answer questions or discuss any issues that they might consider upsetting.

Data were analyzed using constructivist thematic analysis, taking into account the specific organizational contexts in which participants operate, and broader organizational and societal norms, values, and beliefs that shape work experiences and practices. We followed the six phases of thematic data analysis as proposed by Braun and Clarke (2006). We familiarized ourselves with the data by reading and re-reading the transcripts, which led to the development of initial codes. These initial codes were reviewed to identify emerging themes. The themes were then reviewed by both authors across the data set and refined into the three final themes: (a) Institutional coercive control, (b) Cultural and social isolation, and (c) Relational constraints. Our findings with respect to these three themes are discussed next.

## Institutional Coercive Control

Participants in this research spoke of how institutional coercive control, in the form of, for example, barriers to, or denial of, legal protections, housing, social welfare and autonomous residency status, can compound the difficulties of help-seeking and DVSS provision to minority ethnic women.

In Ireland, access to welfare services is dependent on residency rights. This was identified as a first critical issue that all support workers encounter when dealing with migrant victim-survivors. Many such women in Ireland lack any evidence of living in the country. They do not have bank accounts, do not work, and do not own property or hold a lease in their name. They are effectively institutionally invisible. Kate explained how this impacts her ability to support victim-survivors: “She's from [an EU country] (…) she can't prove that she's a habitual resident, because for the last years, she's been technically his dependent. So that just makes that work with her a lot harder because my hands are tied in what I'm able to support her with.”

Participants described encountering victim-survivors with no legal permission to reside in Ireland, with expired visas, or visas tied to the perpetrator. June reported that these women have limited or no access to welfare services and, as a result, are unable to leave their abuser:

What pops up every so often, is if someone doesn’t have their documents, or if they’re on a spousal visa (…), they have such difficulties accessing money or housing or they can’t basically [get any supports] (…). Even if they did want to leave the [perpetrator] they really can’t.

While the DVSS workers acknowledged that, under current immigration guidelines, victim-survivors whose permission to reside in Ireland is linked to the perpetrator can apply for independent immigration status, they emphasized that many are unaware of this provision. However, they reported that others, such as victims of trafficking, applicants for international protection, international students, women with no or expired residency permission, do not fall within this provision. Vicki, a legal officer, noted that non-EU women often don’t leave because they fear deportation. She said they always ask, “If I divorce my husband, can he revoke my visa?” June highlighted that abusers use this fear of deportation to control women, and it was painfully clear from her interview that this not only undermines the effectiveness of supports that she can provide, but also leads her to worry about the very safety of these women: “There's so many of them that are (…) being abused by partners because they know that they can’t report them (…). We can’t just leave women who are being abused on the streets like, we just can’t.”

Faced with situations where survivors have no access to welfare services, support workers are often forced to take actions that they know they probably shouldn’t but feel that they have no choice but to do, if they are to help the victim-survivor. Caroline explained it like this: “We normally don’t. We mind our boundaries …. but we got our job, and I suppose it's upsetting if anything (…). We’re not sticking to our policy (…). Another migrant woman here as well, very severe case and yeah, we would have had to go outside the box for her as well.”

Going “outside the box” means that, when victim-survivors present with additional atypical vulnerabilities, such as being trafficked, victims of torture, or undocumented, support workers feel they must adopt non-standard practices that are really outside of their remit. For example, instead of arranging space in a DV refuge, they may need to advocate for places in homeless hostels or seek charitable support to pay the cost of a B&B. These atypical cases can be difficult to understand, more challenging to manage, and may further strain an already complex and resource-limited service environment. As Sarah shared, the volatility of these situations is upsetting and difficult for the victim-survivors, but also demanding and frustrating for support workers, when they know that the only supports that they can secure are substantially substandard and inappropriate for victim-survivors of trauma and abuse:

I suppose our own emotions can be activated because where our client is going from here is not suitable (…) considering the trauma that they have experienced. It's not considering their medical need. We had a woman who was here (…). She was put into an all-female [homeless] hostel on a top bunk in a room with a very sick child (…). And she lasted four days. (…). We’re seeing more and more women (…) that are coming here. (…). Challenge after challenge after challenge (…) or women who come with that migrant background.

Sarah's quote illustrates how the inability to access appropriate services undermines these women's conviction that they can leave, and this can result in their return to the perpetrator. It also undermines the efforts of support workers who feel powerless to make a meaningful difference to their situation.

DVSS workers explained that many of the resources necessary for survivors’ safety—such as housing, income replacement, and visas–are spread across various state agencies. They noted that the effectiveness of their work often depends on their professional relationships with these agencies and their ability to advocate with these agencies on behalf of service users. They also observed that the nature of advocacy is evolving and that they are losing some of the flexibility they might have had previously. Sarah emphasized that successful advocacy has become increasingly challenging, which is adding to the complexity of their work: “In the past we would have been a bit more successful in supporting women to get the right support from a housing perspective (…). That's becoming more and more difficult to do. So, it does come with complexities.”

The struggles, frustrations, and anger that support workers experience while seeking government support and advocating on behalf of victim-survivors across various agencies are particularly evident in Samantha's account. She highlighted their efforts as they struggle with a system that she feels is just not fit for purpose.

Sometimes [the system] it's not working properly, or you know having to fight (…) to be recognized and have that voice to advocate (…). It's like a game. You have to go to meetings, you have to go here, and you have to network and speak to this minister and that counsellor (…). Yeah, it's quite frustrating.

Most of the organizations represented through the interviews had no legal support worker nor dedicated resources to engage with in-depth legal training. Some participants talked about their struggles to fully comprehend the highly complex immigration landscape and the implications of the wide variety of immigration statuses for their clients. Most acknowledged that they have to refer legal residency matters to specialist organizations, which creates a lot of delays. This puts significant pressure on individual support workers and detrimentally undermines the quality of the service provided. The frustration was palpable in June's comment:

It's too difficult [for support workers]. They can’t know it all. A woman comes and says I have stamp four and they say like, OK. So, what does that mean? Domestic abuse support services can’t know all that (…). They have to get support and help with that so that we can do a good, a better job.

The resource pressures and additional demands imposed on support workers by the institutional challenges and intersectional vulnerabilities of the victim-survivors may adversely affect support workers’ wellbeing, and they are acutely aware of this themselves. As Sarah explained: “The team are very, very aware of that, but for me it's finding the right balance so that (…) they're not getting lost in this vicarious trauma, secondary trauma, and then we're having elements of burnout within that.”

## Cultural and Social Isolation

Support workers emphasized the multiple vulnerabilities experienced by minority ethnic victim-survivor women and described how these vulnerabilities manifest in cultural and social isolation, restricting their help-seeking options and the quality of supports they receive.

Throughout the interviews it was repeatedly recounted that minority ethnic women presenting to DVSSs in Ireland tend to have limited or no awareness of the network of services, rights, and entitlements available to them. Patty noted that they are unfamiliar with the relevant laws and their implications in cases of abuse, of how the court system works, and what to expect from court proceedings. Grace highlighted that raising awareness is a crucial aspect of DV casework, especially when working with minority ethnic women. Rachel similarly emphasized that making them aware of what services exist–should they decide to act–is a tangible first step towards ensuring their safety.

Participants further reported that, when these women do seek help, their interactions with social institutions often become stressful, and so they frequently rely on DVSS providers to accompany them or to advocate for them. While these are acknowledged as core functions of DVSS work, the participants described that advocacy is especially more intense and frequent with minority ethnic women.

The participants reflected that the lack of institutional awareness of available supports is symptomatic of the broader social isolation that most DV victim-survivors face. They observed that this isolation can be so extreme that victim-survivors are unable to function independently and may require assistance with even the most mundane tasks. For example, Samantha described that even women who have lived in Ireland for a significant period, and who may be working professionals, are sometimes unaware of how to perform basic financial transactions:

I can’t believe that she’s a doctor and doesn’t know how to fill in [the online form]. Then we realized it's because there's been isolation. There's been a control by the husband, where the money goes into the bank account (…). But this is common in domestic violence anyway. It's part of the control. But if you're migrant women and you're not in your country, you don’t know what to do (…). The challenges are really far and wide.

Such extreme forms of social isolation require additional support and staff resources, and yet these resources are continuously diminishing across the State (Women's Aid, 2025). The result is that workers struggle to keep up with the demands of their job and readily acknowledge its adverse impact on the quality of the services that they can provide.

Limited institutional and social awareness, combined with isolation, is further exacerbated by language barriers. It is generally recognized that DV perpetrators routinely prohibit their partners from attending English classes as a means of maintaining tighter control over them. Our participants lamented that translators are in very short supply and are not readily available to them. This often forces victim-survivors to rely on friends, relatives, or even their children to help them communicate with support workers. Where this is not even available, support workers resort to using Google Translate to communicate with the women. They universally concurred that the absence of professional translators significantly compromises the quality of interactions. This creates considerable frustrations and challenges on both sides as victim-survivors are unable to fully express their feelings, experiences, and needs, and support workers cannot readily identify or predict their needs. Catherine recounted the struggles that can ensue:

Obviously, it's hard for them. It's hard for us as well because we want to offer them the best support, (…), to offer them the same as for the others (…). By the time that she says it in her language, the other person understands it and then comes back to me and then again it goes back to her. You can see she's frustrated. You could see that she has no patience. You can see that she gets angry from her body language (…). You can feel—it's hard.

Similarly, Sarah argued that difficulties in understanding the victim-survivor can prevent support workers from fully engaging with service users: “They [minority ethnic women] are not met with that empathy or understanding (…). So yes, I think language can be the biggest barrier.” Echoing other participants, Patty further noted the diminution of the quality of supports for this cohort of victim-survivors, compared with other, non-migrant victim-survivors: “I’m not saying it's not effective, but I don’t think it comes across the same.”

Participants believed that this inability to communicate effectively or to recount their experiences in their own words may contribute to victim-survivors feeling devalued, because their authentic voice is not being heard. It likely also compounds their loss of self because victim-survivors of DV routinely have their voices taken away by perpetrators who tell them that no one will believe them.

Operationally, the lack of available professional translators affects the quality of the interactions between support workers and their clients. Grace explained that it impedes support workers’ capacity to fully understand the service user's situation and to propose appropriate solutions. Sarah further noted that “language barrier can be counterproductive (…). We need far more time to empower women.*”* The lack of translation support further affects victim-survivors’ perception of the service and their trust in the support worker's ability to help them.

The low social capital of minority ethnic victim-survivors was a recurring thread throughout the interviews, and it was felt that this significantly compounds the anxieties that they experience. Support workers emphasized how cultural differences may further aggravate feelings of fear and experiences of isolation particularly where societal and religious traditions influence the ways in which victim-survivors are treated by their families and communities. From their experiences, they noted that many minority ethnic women do not want anyone to know that they are being abused. Among the reasons observed by them is the victimization that they face in their communities when they leave the perpetrator. This breaking of social and religious norms leads to shame and embarrassment and often ostracization and even further social isolation. Patty confirmed that many of her clients would not confide in their families. June explained how, in some instances, disclosing intentions to leave an abusive relationship can be particularly dangerous for the victim-survivor:

They are also sometimes afraid, you know, of what their family (…) will say and do if they leave. So, I was speaking with the lady (…). She said that she was a child-bride (…) I wouldn’t have thought she was more than 40. She said that she has [number of] children. Her oldest [child] is 30. And I didn’t even want to ask what age she is because I didn’t want to know. (…). The dowry was paid so she couldn’t get out of this. He used to try to set fire to her. He used to hurt her physically. She showed me scars.

Grace pointed out that the family can often be the source of abuse where there are multiple perpetrators within the household. As a result, many victim-survivors do not risk confiding in any family members.

Support workers emphasized that cultural and religious traditions further affect how DV is communicated and disclosed to them. The following statement from Catherine reflects this, whilst also highlighting how various barriers to accessing support can intersect:

In some of the cultures, they don’t disclose anything, and you have to ask them all the time (…): Have you been sexually assaulted or have you been forced to have sex? (…) You can see that yes, they have, but they don’t disclose that. (…) In some of the cases, it could be culture, it could be religion, it could be, you know, like other things. (…) the barrier is not just the language but loads of the other factors.

Minority ethnic women's cultural distrust of formal authority was an issue perceived by the participants, and something that impedes their work. They highlighted that trust is an essential component of the interpersonal relationship that they establish with victim-survivors. It also acts as a conduit to facilitating access to DVSSs and engagement with other service providers, such as social welfare officers, judges, or police. Grace pointed out that “The trust needs to outweigh the fear for them. To really, you know, engage and for us to be able to use an intervention.” Patty explained that an important aspect of working with minority ethnic women is to try to normalize help-seeking behaviors. She noted that most minority ethnic women are terrified to contact the police or go to court, and in some cases, the fear of interacting with state agencies discourages them from ever seeking help.

## Relational Constraints

The support workers all spoke of the necessity to adopt the individual victim-survivor's perspective, or to relate to them, in order to deliver what they termed as a quality survivor-centered service that is non-judgmental such that the victim-survivors feel heard and believed. They were strongly of the view that this type of service must be driven by the victim-survivors’ needs and highlighted that support workers are ever vigilant to ensure that any action taken on behalf of the service user aligns with what the women want to do. However, they also emphasized that providing such services is not always possible due to relational constraints imposed by systemic institutional barriers and intersectional vulnerabilities.

Adopting the victim-survivor's perspective requires anticipating the practicalities of interactions. Participants spoke about creating a friendly and welcoming atmosphere in meetings, adhering to an informal dress code to distance the service from formal bureaucratic institutions, and knowing what to say and when to say it. These basic interactional practices help them establish and maintain trusting relationships with service users and are intended to signify a supportive, as opposed to a directive, role. As Catherine underlined, an inability to adopt the victim-survivor's perspective renders support workers unable to offer them appropriate services: “Like you can't offer them professional support if you're not trying to walk in their shoes.”

Kate emphasized the importance of understanding the victim-survivor's needs and acknowledged that support workers must be careful to ensure that their own perceptions and beliefs do not influence the victim-survivors’ decisions. She underscored the importance of exhibiting empathy in dealing with DV cases: “It's good for them to see that I can relate to what they’re going through. Because I think if you’re experiencing this, you want the person that you’re talking to to understand that (…). So, I think it kind of just (…) makes us human.”

There was evidence that participants not only identified practical needs, or engaged in this cognitive role-taking, but also engaged in “relating,” or seeking to anticipate how survivors felt. In addition to trying to understand the victim-survivor's perspective, they also reported incidences of sharing and responding to their emotions—empathetic role-taking. They recalled that empathizing helps them to alleviate some of the victim-survivor's distress. Patty suggested that adopting an individual victim-survivor's perspective is critical to ensuring their safety: “It's very easy for us to sit down and say, oh, you need to go to court, and you need to do this. But you have to understand, that for some women that might make things 100 times worse.”

The interview data suggest that a combined approach of understanding practical needs (cognitive role-taking) as well as relating to emotional needs (empathic role-taking) is considered crucial for successful interactions, effective casework, and the overall quality of the experience that victim-survivors have when engaging with DVSSs. That said, support workers expressed their worry about being able to provide an adequate survivor-centered service to some minority ethnic women. They cited the systemic and institutional barriers that limit the range of options they have available to them, and which create confusion around rights and entitlements. Kate underscored how the inability to communicate with minority ethnic women can impede emotional support and limit support workers’ ability to build trusting relationships: “You know you can’t really do the emotional support piece.” She admitted that in the face of intersectional vulnerabilities, such as when victim-survivors do not speak English and no translator is available, she is unable to offer them emotional support and instead focuses on the practical challenges of their case: “I mostly do practical stuff with them.”

Moreover, participants identified the additional socio-cultural challenges that require greater effort on their part to understand and empathize with their clients. Faced with additional vulnerability, June argued that support workers need to understand the cultural specificities of the victim-survivor as they anticipate their needs because failure to do so can be detrimental to the victim-survivors:

Like I’m blue in the face saying to people (…) if someone comes to you and says that she's afraid of her family, don’t send her home (…). If someone comes to you, a young girl comes and says: I am married to this guy. I’ve been married to this guy. Maybe forcefully or maybe arranged or whatever. He's hurting me. I don’t know what to do. And they say—I’ll call your mum. And I said you can’t do that (…). I don’t think anybody is really prepared for that yet. So that is the next step. We need to talk about this before we have killings.

Participants argued that, to understand and relate to minority ethnic victim-survivors, training that focuses specifically on how to support them, including cultural awareness, is vital. However, as Ella emphasized, such training is infrequently available: “No, [I haven’t come across one], not yet. I am attending one soon for Ukrainian women experiencing domestic abuse. But that (…) would be my first one.” Speaking about the need for this type of training, Grace argued: “If you are not trained in what you’re dealing with, how can you risk assess and, without risk assessing, they're slipping through the net.”

Combined, the stories recounted by our participants point to overwhelming evidence that many minority ethnic victim-survivors in Ireland face systemic and institutional barriers and personal vulnerabilities that impede access to basic safety nets. As reported here, these barriers and vulnerabilities intersect to compound difficulty both in receiving help and in providing critical DVSSs. In some cases, the systemic and institutional failures force the victim-survivor back to the abuser. These intersectional vulnerabilities clearly impact the quality of interactions with frontline support workers. Support workers highlighted the lack of ethnically inclusive and culturally responsive training, which makes it more difficult to fully understand and anticipate these women's individual circumstances and needs. As a result, the delivery of survivor-centered DVSSs is constrained. Moreover, the key ingredient to quality casework–empathy through understanding–is often absent, as workers struggle to understand and then adopt the victim-survivor's point of view.

## Discussion

As indicated at the outset, DV is a form of gender- and sexual-based violence, and common definitions of DV focus on abuse perpetrated by an intimate partner or family member. However, our research revealed that DVSS workers assist not only victim-survivors of partner or familial abuse, but also victims of human trafficking, torture, and those affected by multiple abusers, broadening the conventional understanding of DV. The multiplicity of intersectional vulnerabilities associated with minority ethnic status requires going “outside the box” to effectively support these victims.

Research has long argued that immigration status, as a mechanism of control, is linked to the exacerbation of gender-related vulnerabilities (Erez & Britz, 2006; Lundy & Crawford, 2025; O’Neal & Beckman, 2016; Voolma, 2018) and poses a critical risk to minority ethnic women who present to DVSSs. Migrant victim-survivors with precarious immigration status (including those undocumented, trafficked, or on spousal or with expired visas) are often in situations where their residency and associated social security rights depend on maintaining a relationship with the perpetrator–such that, “if the woman is in this country illegally, and the husband is legal, he will do whatever he wants with her, because she is at his mercy” (McIlwaine & Evans, 2020, p. 106). Many of these women are socially isolated, do not speak English, and have no social networks beyond those of their partner. As a result, they are completely reliant on their intimate partners, families, and diasporas, all of which exacerbate their safety risks (Reilly, 2023). The fear of deportation or repatriation limits their ability to leave the abuser/s, discourages help-seeking, and restricts the range of supports available to them–often to an absolute minimum. Our findings lend credence to Voolma's (2018) argument that as long as the State continues to deny minority ethnic women access to social services due to their immigration status, its primary objective remains border control–rather than the ending of violence against all women, regardless of her status.

Elhelw (2024) observed that if intersectionality speaks to how different positionalities collide in the lives of women, then it must also speak to the need for diverse systems of support. As our research showed, this intersectional perspective highlights the multifaceted and mutually reinforcing dynamics of control, exclusion, and isolation that converge in the domestic abuse experiences of minority ethnic women, resulting in multiple, overlapping forms of discrimination and barriers to support, and limiting their help-seeking options. Survivors present with precarious legal status, financial insecurity, inability to find work, different language, culture and religion, extreme social isolation, few if any support systems and some are victim-survivors of human trafficking or torture. These factors change the typical dynamics of interactions between support workers and survivors and affect the quality of casework.

Our findings point to a significant and systemic failure of existing DVSSs in Ireland to adequately address the interlocking, intersectional needs of minority ethnic women who seek help and, in some instances, serve to discourage victim-survivors from engaging with support systems altogether. As our research shows, their journey to safety is complicated by systemic and institutional barriers and intersectional vulnerabilities, making that journey particularly perilous and challenging. Confirming Sokoloff and Dupont's (2005) findings, our participants described how institutional coercive control, most notably linked to immigration status but with cascading effects, undermines victim-survivors’ autonomy and independence, often forcing them to remain with their abuser. Our findings also confirm those of SI (2023), revealing that the system in Ireland bluntly (and sometimes brutally) prevents so-called “undeserving” applicants from accessing State resources, including welfare services that are desperately needed in cases of abuse. As Catherine observed, many of those who design the system do not understand the dynamics of DV “at all.”

A recurring theme throughout the interviews was the profound sense of purpose and validation that support workers experienced in enacting their roles. The sharing of intimate stories and the trust placed in support workers were recognized as privileges. To provide a deeper and more nuanced understanding of how systemic, institutional and intersectional challenges affect the ways in which DVSS practitioners carry out their work, our research adopted a role-taking perspective (see, e.g., Groggel, 2023). This additional lens helped us capture how the development of a strong trust-based relationship between DVSS workers and their service users is considered a foundational aspect of their interactions because, as Grace put it, it needs to “outweigh the fear”’ so that service users feel safe to engage with supports.

Groggel (2023, p. 50) emphasizes that role-taking helps support workers to “gather relevant details, since traumatized clients often struggle to align their narratives in a chronological or coherent form.” Repeatedly, and clearly reflecting cognitive role-taking, our participants spoke about the need to adopt the victim-survivor's perspective to comprehend service users’ individual experiences of abuse, anticipate their needs, and propose appropriate solutions. They reflected on the experiential knowledge they had acquired, which enabled them to understand the immense strength and courage it takes for these women to come forward and disclose abuse. This role-taking lies at the heart of how DVSS practitioners approach their work. As Sarah described, they take on the responsibility of sharing in her journey to safety and, together with the victim-survivor, strive to help her live a life free from abuse.

To engage in role-taking, support workers must comprehend another's position, read social cues, interpret service users’ individual circumstances, and anticipate their needs accordingly (Groggel, 2023). However, our research found that effective cognitive role-taking was often impeded, primarily by systemic institutional barriers that hindered practitioners’ ability to do their jobs effectively. Participants expressed ongoing frustration with the time and effort required to secure even the most basic supports for these socially marginalized women, and with the various struggles they face in navigating the complex institutional systems that limit access to essential services. Referring to residency requirements, Kate noted the limited scope of supports available to some minority ethnic women, which, as Sarah recounted, posed one challenge after another. As June underscored, some minority ethnic women face such significant barriers and exclusion that, even if they want to leave their abuser, they essentially cannot. Despite feeling overwhelmed at times and often unable to offer these women safety, which critically limits their capacity to engage in role-taking, support workers frequently go beyond organizational protocols to ensure that no victim-survivors of abuse are left destitute and without shelter. As our data show, these struggles are not without consequence for the support workers themselves as they exhaust not only their practical resources but also the emotional ones.

In addition, by combining intersectionality lens with role-taking, our study reveals the hidden relational dimensions and barriers to effective DVSS provision. We clearly show that culture, isolation and language pose additional constraints that impact both cognitive and empathic role-taking because they frustrate the establishment of that critical initial interpersonal interaction. Incomplete understanding of the cultural and social contexts of the victim-survivors may hinder support workers’ capacity to adopt their perspective as, due to trust issues, cultural discourse and rules, or embarrassment, some are reluctant to share information about their trauma. Language barriers pose another obstacle to relationship-building and effective interactions. Resorting to Google Translate or having a child translate the conversation significantly disrupts the interactional dynamic between the support worker and victim-survivor and impedes engagement in both aspects of role-taking. For instance, as Kate admitted, because the demands of casework are so high with some minority ethnic victim-survivors, she cannot engage in empathetic role-taking and instead focuses only on overcoming the practical challenges so that she can offer some help to them. Finally, to better understand and relate to this cohort of service users, support workers underscored the need for the nationwide availability of ethnically inclusive and culturally responsive training. As Grace pointed out, the inability to comprehend the intricacies of minority ethnic women's experiences means that “they’re slipping through the net.”

Together, the results point to a fragmented and strained DVSS system in Ireland that is inadequately equipped to meet the intersectional needs of minority ethnic women. Participants recounted how these intersectional vulnerabilities alter the typical dynamics of interactions between support workers and survivors, and they believe this adversely affects the quality and effectiveness of their work. They advocate for a victim-survivor-centered approach to DV casework–one that acknowledges the multidimensional barriers to safety. Additionally, they call for improved connectivity and collaboration between various DVSS providers and State agencies as well as the allocation of appropriate resources to dismantle existing barriers to service provision in Ireland. This, they believe, will help to ensure that all victim-survivors of DV have access to high-quality DVSSs and that no one, regardless of their immigration status, culture, language or any other intersectional vulnerabilities, is left behind.

## Conclusion

Intersectionality represents a powerful lens through which to understand the multiple and mutually reinforcing systems of control, exclusion, and isolation in the DV experiences of minority ethnic women. Role-taking presents a useful mechanism to interpret how intersectional challenges filter through into the ways in which DVSS practitioners complete their work. Together, intersectionality and role-taking offer a holistic and nuanced understanding of how the systemic and institutional shortcomings within DVSS impact both victim-survivor and support worker. In so doing, they give expression to an urgent need to develop new initiatives that are responsive to intersecting systems and structures of oppression and exclusion, and which will enable the tailoring of supports that are appropriate for all victim-survivors of DV. The aim of our research was to uncover support workers’ experiences of DVSS provision to minority ethnic women as situated within the broader context of the DVSS landscape and the wider “socio-cultural and structural conditions” (Braun & Clarke, 2006, p. 85). The combined evidence presented in this article points to a web of compounded systemic, institutional and intersectional challenges that substantially affect both the quality of supports available to minority ethnic women who experience DV in Ireland and constrain service providers’ capacity to understand and identify with these victim-survivors’ experiences. We argue that an intersectional, culturally sensitive and human rights-informed support service response to DV is now required so that minority ethnic women in Ireland who experience or are at risk of experiencing DV receive a continuum of required supports from the key DVSS providers. Exploring the experiences of these women from a combined intersectional and role-taking perspective enables a better prospect of identifying required policy and practice enhancements to support provision.
